# Prospective Memory Impairments in Schizophrenic Patients

**Published:** 2014

**Authors:** Imanollah Bigdeli, Azin Farzin, Siavosh Talepasand

**Affiliations:** 1 Associate Professor, Department of Clinical Psychology, School of Psychology and Educational Sciences, Semnan University, Semnan, Iran.; 2 Department of Clinical Psychology, School of Psychology and Educational Sciences, Semnan University, Semnan, Iran.; 3 Associate Professor, Department of Educational Sciences, School of Psychology and Educational Sciences, Semnan University, Semnan, Iran

**Keywords:** Prospective Memory, Retrospective Memory, Schizophrenia

## Abstract

**Objective:**

Memory impairment is one of the most pervasive cognitive dysfunctions in schizophrenic patients. The aim of the current study was to conduct the most comprehensive assessment of how prospective memory (PM) is affected in schizophrenia in comparison with healthy controls.

**Methods:**

In this study, 30 first-episode schizophrenic patients who fulfilled the diagnostic criteria of the Diagnostic and Statistical Manual of Mental Disorders based on the diagnostic interview were recruited from eight regional psychiatric clinics in Iran. All participants were males (age 27-42). Moreover, 28 healthy controls were recruited from the same social-class as the patients. The Prospective and Retrospective Memory Questionnaire (PRMQ), PM tasks, and the Virtual Week Board Game were administered. Moreover, clinical symptoms were rated using the positive and negative symptoms scale.

**Results:**

The results showed that in all of the memory types, the group with dominant positive symptoms was superior to the group with dominant negative symptoms. In addition, the results showed that in all of the memory types, the control group had superiority to the schizophrenic group. The most considerable differences between groups were in time-based PM tasks, irregular event-based virtual week tasks, and retrospective tasks (PRMQ).

**Conclusion:**

The current study confirmed that schizophrenic patients have severe PM deficits.

## Introduction

Cognitive deficits are considered a key feature of schizophrenia. Although many schizophrenic patients respond to treatments, their cognitive deficits are long-term and can lead to poor social and occupational outcomes (1). Memory impairment is one of the most pervasive cognitive dysfunctions in schizophrenic patients (2-4). Most previous studies on memory impairments in schizophrenia have been focused on retrospective memory (RM) (5-7). Nonetheless, there are few studies that have focused on the nature and degree of prospective memory (PM) deficits among this clinical group (8).

PM is one of the most essential components of daily life, because it literally remembers to remember. PM is an aspect of episodic memory that involves the construction, maintenance, and the execution of future intentions and has significant implications for daily functioning (9).

The main characteristics of PM include: a) a delay between encoding and running the intended action; b) during the delay, the individual has to be engaged in an ongoing task; and c) there is no external reminder for the intended action. Hence, compared to RM, there is more demand on self-initiation in PM (10).

Furthermore, PM can be divided into 3 types based on the nature of the associated cue and the future intention; time-based, event-based, and activity-based (11).

Hypothetically, PM depends upon the integrity of multiple cognitive abilities associated with frontostriatal and temporolimbic systems (12), including executive functions, working memory, episodic RM, and information processing speed (13).

The cognitive impairments caused by schizophrenia are numerous and disabling (14-17). Schizophrenia is associated with mild-to-moderate impairment in several cognitive domains, including attention, language, executive functions, and memory (14, 16, 18). Emerging evidence indicates that individuals with schizophrenia may exhibit deficits in PM, a dissociable and ecologically important aspect of episodic memory entailing the formation, maintenance, and execution of future intentions (19).

Moreover, schizophrenic patients have functional connectivity impairment suggesting impairment in the frontal lobes, temporal lobes, and other areas (20). Based on these findings, it is logical to assume that individuals with schizophrenia have PM impairment (21-22). Realizing the nature and the extent of PM deficits in these patients would provide crucial information about their behaviors and help in finding the most efficient management and rehabilitation (22).

Until the present day, only a few empirical studies on PM in schizophrenic patients have been published. Some previous studies have found that event-based and time-based PM were impaired to the same degree (6, 23), whereas others have shown that time-based PM is significantly more impaired than event-based PM (23, 24). The duration of illness and demographic variables cannot be well understood as the results of previous studies were inconsistent. For instance, some studies found significant relationships between PM and dominant negative symptoms (23, 25), but some others did not (26, 27).

Thus, clarifying these relationships is important for understanding the nature and the extent of PM impairment in schizophrenic patients. Findings that clarify the nature of PM impairment in patients with schizophrenia are not only crucial for schizophrenia management, but also for disease prevention in terms of identification of vulnerable individuals and gene candidates. Thus, the main aim of the current study was to conduct a comprehensive assessment of PM deficits (the nature and the extent) in schizophrenic patients, by investigating different types of PM failures observed in their performances.

## Materials and Methods

The convenient sampling method was used because of the nature of the study; the authors aimed to find as many first-episode schizophrenic patients as they could at the time of the study.

A total of 31 episodic schizophrenic patients who fulfilled the diagnostic criteria of the Diagnostic and Statistical Manual of Mental Disorders (DSM-IV-TR) based on diagnostic interviews (using the structured clinical interview for DSM-IV-TR) were recruited from 8 regional psychiatric clinics in Mashhad, Iran, during April to May 2010 (28). All participants were males, and they were between the ages of 27 and 42. Patients with a history of neurological illness or drug dependence (according to clinical records, and information from the interview with the patients and/or their families) were excluded. Clinical symptoms were rated using the Positive and Negative Symptoms Scale (PANSS) (29).

Moreover, 28 healthy controls were also recruited from the same social-class and neighborhood as the patients. Furthermore, the control and study group participants were matched in terms of age, educational level, and social class. None of the healthy controls had family history of psychiatric illnesses, suffered from a neurological illness, or had a history of drug abuse/dependence. The study was approved by the Ethics Committee of the School of Psychology, Semnan University, Iran, (17 March 2010; 98/88/2051).

To measure both objective and subjective PM performance of the participants, this study has used two types of PM test.

***Subjective measures of PM***

The Prospective and Retrospective Memory Questionnaire (PRMQ) was used to examine the subjective measures of PM. The PRMQ is a 16-item questionnaire. Each participant was asked to rate the frequency of occurrence of each type of memory failure in their daily life on a 5-point scale. Confirmatory factor analysis indicated that the PRMQ consists of a general memory factor together with additional prospective and RM factors among a group of the general adult population (30).

The reliabilities were also acceptable (alpha = 0.80-0.98), and the demographic variables were not found to influence ratings (31). The Farsi version of the questionnaire was used for the current study. In the current study, this version has been shown to have an impressive internal consistency (Cronbach's α = 0.97) and test-retest reliability (r_tt_ = 0.73).

***Objective measures of PM***

Virtual Week Board Game: Virtual week has been used to assess PM (32). Virtual week is a computer-based board game, in which the participants move around the board with the roll of the dice. As participants move around the board, they must make choices regarding their daily activities and remember to do some activities that are similar to their daily activities (PM tasks). Each day of virtual week includes 8 PM tasks (4 regular, 4 irregular), which allows the identification of any type of PM impairment. The Farsi version of the questionnaire was used for the current study. In the current study, this version has been shown to have an impressive internal consistency (Cronbach's α = 0.74). In addition, the split-half reliability of the virtual week (not the computer-based version) was estimated to be 0.90 for the overall measure, and 0.79, 0.65, and 0.77 for the regular, irregular, and time-check tasks, respectively.

PM-tasks: The computer-based “PM-tasks” has 3 subtypes; event-based, time-based, and activity-based tasks. Ongoing tasks are divided into semantic and perceptual tasks. In the semantic event-based PM task, a 4-character phrase is presented in the center of the screen, and the participants are required to judge whether the phrases are idioms or not.

The semantic time-based PM task is the same as the semantic event-based PM task except that a clock is placed at the upper right side of the screen and the participants are required to monitor the time throughout the session. The perceptual time-based PM task is the same as the perceptual event-based PM task except that a clock is placed at the upper right side of the screen and participants are asked to monitor the clock and press the spacebar every 1 minute. The Farsi version of this tool was used in the current study, this version has been shown to have an impressive internal consistency (Cronbach's α = 0.85).

The PANSS is a 30-item (7 items for positive and 7 for negative symptoms, and 16 items for general psychopathology), 7-point rating instrument which has adapted 18 items from the brief psychiatric rating scale (33) and 12 items from the psychopathology rating scale (34). The Farsi version of the PANSS was used for the current study. This scale has been shown to have an impressive consistency (r = 0.99).

All the tests were carried out in a room designed for conducting this study in 2 sessions of morning and afternoon. First, the PRMQ (which is a pencil-paper questionnaire), and then, the PM computerized tasks were carried out. The 4 PM-tasks were given in the following order: semantic time-based, perceptual event-based, semantic event-based, and perceptual time-based. The Virtual Week was used to measure PM in the form of some activities similar to daily activities. Finally, the participants were interviewed, and rated using the PANSS.

## Results

The mean and standard deviation of all the variables was reported in table 1. In patients with dominant positive symptoms, the mean PM and all Virtual Week scales were higher than the scores of patients with dominant negative symptoms (mean difference = 0.99). In PRMQ, as was expected, the results showed the opposite. In Virtual Week, maximum and minimum differences were observed in irregular time-based and irregular event-based tasks, respectively.

T-test with Bonferroni correction was used to compare schizophrenic patients with dominant PANSS. The results showed that in all memory types, schizophrenic patients with dominant positive symptoms were more successful than those with dominant negative symptoms (Table 1).

Furthermore, t-test with Bonferroni correction was used to compare controls and schizophrenic samples. Results demonstrated that in all memory types, the control group was more successful compared with the schizophrenic group (Table 2). Most of the differences were found in time-based PM tasks, irregular event-based Virtual Week tasks, and retrospective PRMQ tasks.

## Discussion

The purpose of the present study was to investigate PM, its different components, and the relationship between PM and clinical symptoms of schizophrenic patients (compared with the control group). The results suggested that schizophrenic patients, compared with normal participants, had lower success rate in PM. This result was consistent with similar studies which have found that PM performance of the general population is more successful than schizophrenic patients (6, 23-25, 27, 35-37). In addition, this result was consistent with that of neuropsychology studies that have reported that PM impairment is related to deficit in the frontal lobe (38).

Based on the results of the present study, it has been demonstrated that the performance of schizophrenic patients in time-based tasks is less successful than event-based tasks of PM. This result is consistent with that of some studies (24, 27), which have reported less successful time-based PM performance in comparison with event-based PM performance in schizophrenic patients. However, its results are inconsistent with other studies that have reported the same level of impairment for schizophrenic patients in time-based and event-based tasks (6, 23, 39), because this study found that schizophrenic patients were less successful in time-based than event-based PM performance. A possible explanation for this inconsistency is the essence of time-based and event-based tasks. Event-based tasks that were presented in this study provided recognition cues that were more practical, but such similar conditions have not been used for time-based tasks.

**Table 1:** Comparison of memory impairments between schizophrenic patients with dominant positive and negative symptoms

**Variables**	**Positive symptoms (n = 17)**		**Negative symptoms (n = 13)**		**Mean difference** *
	**Mean ** **± ** **SD** †		**Mean ** **±** **SD**†		
PM‡ (total)	-0.37 ± 0.22		-1.36 ± 0.34		-0.986**
PRMQ§ (total)	-0.76 ± 0.31		-1.09 ± 0.21		-0.325**
Virtual week					
Regular event-based	-0.77 ± 0.17		-0.37 ± 0.10		-0.393**
Regular time-based	-0.66 ± 0.23		-0.30 ± 0.14		-0.357**
Irregular event-based	-0.61 ± 0.17		-0.27 ± 0.14		-0.340**
Irregular time-based	-0.66 ± 0.22		-0.23 ± 0.14		-0.426**
Irregular beginning	-0.64 ± 0.19		-0.26 ± 0.11		-0.379**
Irregular during	-0.64 ± 0.15		-0.25 ± 0.14		-0.387**

* All mean differences were statistically significant,

** P < 0.05;

† Standard deviation;

‡ Prospective questionnaire;

§ Prospective and retrospective memory questionnaire

**Table 2:** Comparison of Memory Impairments in schizophrenic patients (n = 30) and control group (n = 30)

**Variables**	**T**	**df** †	**Mean difference**
PM‡ scales			
Event-based	-09.681	58.000*	-1.466**
Time-based	-12.093	40.186*	-2.450**
Activity-based	-14.316	29.000*	-0.483**
Total	-13.664	43.698*	-1.605**
Virtual week scales			
Regular event-based	-07.493	34.002*	-0.353**
Regular time-based	-07.741	37.710*	-0.403**
Irregular event-based	-11.779	30.757*	-0.510**
Irregular time-based	-08.192	33.280*	-0.446**
Irregular beginning	-09.559	34.228*	-0.453**
Irregular during	-11.089	30.789*	-0.503**
PRMQ‡ scales			
Prospective and retrospective	-17.439	58.000*	-16.460**
Prospective	-12.843	58.000*	-7.460**
Retrospective	-16.736	58.000*	-09.00**
Short term	-17.179	58.000*	-08.93**
Long term	-14.673	58.000*	-07.53**

* df was adjusted for unequal variances assumption,

** P < 0.01;

† Degree of freedom;

‡ Prospective questionnaire;

§ Prospective and retrospective memory questionnaire

In addition, the present study showed that schizophrenic patients, in comparison with the general population, had less successful performance in all PM tasks. This result was consistent with previous findings (6).

This study showed that schizophrenic patients with dominant positive symptoms had better performance in PM tasks in comparison with the patients with dominant negative symptoms. These results are consistent with some studies that have reported that schizophrenic patients with severe social withdrawal are unable to remember external cues and convert their goals into actions (17, 23). However, these results were inconsistent with some studies that have shown no relation between patients' performance and their symptoms (23-26). A probable explanation is that in this study all of the patients were first-episodic patients, who have never taken any medications for their condition or been hospitalized, whereas the previous study subjects were on medication and/or had been hospitalized before or during the study.

Other findings of the present study showed that objective and subjective deficits of schizophrenic patients were significantly different. Some researchers reported that schizophrenic patients' performances were less successful than the control group in all PM task types (27). This result was consistent with the finding of the present study; schizophrenic patients had less successful performance in computerized tasks in comparison with the control group. Moreover, their performance in computerized tasks differed from that in subjective tasks. Chan et al. reported that schizophrenic patients have no insight and cannot report deficits of their subjective PM (35). The present study showed that schizophrenic patients, in comparison with the control group, had poorer performance in subjective tasks. Therefore, there is no evidence to confirm the reports of Chan et al. (35). However, different samples of schizophrenic patients could be a probable explanation for this inconsistency.

The first limitation of this study could be that the participants were recruited from only 8 regional psychiatric clinics in Mashhad, Iran. Hence, the findings may not be generalized to other regions of Iran. The second limitation is related to research design. The present study design is ex-post facto; therefore, we cannot make a causal inference from the results. Finally, one of the most important limitations of this study was the use of male participants alone. 

## Notes

**Declaration of interest:** None.
